# Neonatal Pictures in a NICU as a Mode of Nursing Intervention to Enhance Parent-Infant Bonding: Parents’ Experience during the COVID Pandemic

**DOI:** 10.3390/ijerph20043576

**Published:** 2023-02-17

**Authors:** Anna Aftyka, Beata Rybojad, Wioletta Mędrzycka-Dąbrowska

**Affiliations:** 1 Department of Anaesthesiological and Intensive Care Nursing, Medical University of Lublin, 20-081 Lublin, Poland; 2 Clinic of Anaesthesiology and Paediatric Intensive Care, Medical University of Lublin, Gebali Str. 6, 20-093 Lublin, Poland; 3 Department of Anaesthesiology Nursing & Intensive Care, Faculty of Health Sciences, Medical University in Gdansk, 80-211 Gdansk, Poland

**Keywords:** parents, newborn, pandemic, NICU

## Abstract

Introduction: Neonatal departments around the world have changed their policies to prevent the spread of infection during the COVID-19 pandemic. The birth of an extremely premature baby can disrupt physical contact between the mother/parent and the baby. This situation affects the bonding process between mother and child. The aim of the study was to investigate the opinions of parents who receive photographs or videos of their children electronically on the usefulness of this intervention, as well as the emotional reaction of parents to the photos or videos received, and potential ways to improve the intervention. Methods: The study used a qualitative approach and relied on phenomenology, which is a research method used to study experience as experienced from the subjective point of view. Pilot interviews were conducted in January and February 2021, and the final study ran from March to June 2021. Results: The uploaded photographs and videos provided a useful communication tool. The parents’ emotions at the proposal to send photographs of the child and while viewing the first photographs were strong and marked by considerable ambivalence. Conclusions: This study showed how important it is to ensure communication between the parent and the medical staff. Despite the positive reception, in the future obtaining consent from the legal guardian for taking pictures should be considered, whether this form will be accepted, and to ensure the presence of medical staff while the parent is watching the photographs/videos, as this method of communication will not fully ensure direct skin-to-skin contact to build bonds between the parent and the infant. Neonatal intensive care units need to find strategies to mitigate the impact of separation on parental experiences and bonds should a similar situation arise in the future.

## 1. Introduction

An infant’s admission to a neonatal intensive care unit (NICU) inevitably causes the parents emotional stress. The deepest relationship between a mother and her unborn child begins in utero through subconscious co-conditioning of the pairs’ autonomic nervous systems. This incredible early learning is the primary factor that helps the neonate to form emotional relationships with a mother and a father [1]. Future parents are looking forward to the birth of their child. The first contact between a mother and her baby is crucial for future bonding. Immediate skin-to-skin contact and breastfeeding within two hours after birth are irreplaceable. They make mothers more sensitive to a newborn’s needs [2,3].

Epidemiological limitations cause a change in the work organization of many health care units and impose limitations on the contact between parents and their newborn children treated in NICUs. If something goes wrong, and a neonate is born much too early or in a severe condition, he (or she) needs to stay in hospital longer than he (or she) is supposed to. Parental contact is a well-established component of care for critically ill infants, and parents used to be encouraged to visit neonatal intensive care units (NICUs). Family-centered care (FCC) used to be a normative practice in NICUs all over the world [4]. Bonding is crucial to the developmental process no matter whether a baby is healthy at home or sick in the NICU. Postpartum separation disrupts the early physiological/emotional parent–infant relationship [2]. The bonding strategies include, among others, skin-to-skin contact, touching, massaging, talking and singing to the baby. It is important particularly for the brain because synaptogenesis, dendritic growth and neuronal differentiation take place [5]. If a neonate is stable enough (even intubated and mechanically ventilated), it is possible for a parent to hold and handle the baby. This makes an extreme difference. The worst experience is when parents are not able to hold their baby before the baby dies. Besides the direct contact with neonates, physicians describe them to their parents and encourage them to visit as often as possible taking into account all the restrictions [6].

### 1.1. Organization of Medical Care in Neonatal Intensive Care Units during a Pandemic

Neonatal units across the world have altered their policies to prevent the spread of infection during the COVID-19 pandemic [7]. In the spring of 2020, NICUs closed for parents and other family visitors. In our NICU, it is even harder because we are separated from obstetric departments and neonates are transported from many different hospitals without their mothers. There was no option for parents to stay overnight even before the pandemic because the NICU is a part of the Pediatric Anesthesiology and Intensive Care Unit and consists of one big room for eight or nine neonates and two rooms for babies requiring separation (today used for COVID-19 patients as well). Parents could visit their children routinely during daytime, and in individual severe cases, any time and as long as it was necessary (a dying child, urgent surgery to save a life, etc.). Most of the parents ask for a baby baptism and, if there is enough time, a hospital chaplain performs that. Parents are generally present at this short ceremony. Very soon a decision was made to communicate with the parents via long telephone conversations with our clinical psychologist, who also has a medical degree. During the first talk, parents expected more than just to hear about their babies—they would also like to see them. Therefore, taking photographs and mailing them after a writing consent has begun. Subsequently, a psychologist received a separate cell phone with a recording function and started sending short films to the parents. In the meantime, vaccinations for parents of preterm babies were introduced and this at least allowed them to come every day for 1–2 h (depending on current occupancy in the ICU). Parents who are not vaccinated need to perform a COVID-19 test (which is valid for 72 h) or show a document that proves they have had COVID-19 in the past six months. Due to the pandemic situation, in April 2020 an intervention aimed at strengthening the bond between mother and child was initiated and implemented in the ICU of the University Children’s Hospital in Lublin which involved regularly sending photographs of the child to parents by email.

### 1.2. Aim

The aim of the study was to investigate the opinions of parents who receive photographs or videos of their children electronically on the usefulness of this intervention, the emotional reaction of parents to the photographs or videos received, and potential ways to improve the intervention.

## 2. Methods

### 2.1. Study Design

The study used a qualitative approach and relied on phenomenology, which is a research method used to study experience as experienced from the subjective point of view. Phenomenology, developed by Edmund Husserl, deals with how things are manifested in the experience of individuals, how people perceive and how they talk about objects or events, rather than describing a phenomenon according to a predetermined categorical, conceptual system and scientific criteria [8].

The study used an in-depth, semi-structured individual interview with parents of newborns hospitalized in the intensive care unit during a pandemic, describing their experiences of responding to an intervention involving sending photographs or videos of their children.

Pilot interviews were conducted in January and February 2021, and the final study ran from March to June 2021.

### 2.2. Sample and Recruitment

After the child was admitted to the Intensive Care Unit, the parents were asked if they would like to receive photographs of their child electronically within three days and were asked for a written confirmation of their consent to send photographs of the child. Such a desire was declared by the vast majority of parents—about 95%. Before the photographs were sent, the parents were informed about the child’s appearance, the medical equipment visible in the photographs and its purpose. The most common were: tracheal tube, gastric probe, ECG electrodes, pulse oximeter sensor, intravenous access. Simplicity and comprehensibility of the message were taken care of and medical nomenclature was avoided. The endotracheal tube was called the breathing tube, the gastric tube—the tummy tuck tube, or the food tube, etc. The non-medical aspects of the child’s appearance were emphasized, e.g., hair color, facial expression, physical activity, etc. The photographs were taken by amateur members of the therapeutic team using a mobile phone without opening the incubator. Next, the photographs were emailed via business email. Photographs (an average of five shots a day) and shorts films (10–30 s) were sent to the parents twice a week, on Mondays and Thursdays. The photographs were not subjected to photo-processing except for cropping in selected situations. As far as possible, a favorable appearance of the child in the photograph was taken care of by selecting the appropriate lighting and selecting the frame (portrait photograph, silhouette photograph, etc.).

### 2.3. Characteristics of the Researcher

The interviews were conducted by an experienced registered nurse. All participants had prior personal and/or telephone contact with the researcher during the hospitalization of their child in the NICU due to her work in the position of clinical psychologist and were informed about it. The researcher told the participants that the practical effect of the research was to identify ways to improve the quality of support provided to parents of children treated in intensive care units by a therapeutic team.

### 2.4. Participant Selection

The selection of participants was intentional—those invited to participate in the study were parents of newborns of different period and course of treatment, with a variety of health problems (e.g., extreme prematurity, severe asphyxia at birth in a full-term infant, surgical problems, such as atresia of the esophagus, sepsis, respiratory failure) and different prognoses. Potential participants in the study were recruited after a personal interview during their visit to their child or by telephone, in the course of the standard care of the family of a child treated in an ICU after stabilization of the child’s health. Sixteen parents participated in the study; none of the invited participants refused to participate. Due to the inclusion criteria, six parents were not invited to take part in the study, due to the unstable condition of the child, which could generate a high risk of causing increased mental suffering.

### 2.5. Questionnaire Development

The interview was to focus on five key questions:

1. What do you think about the idea of sending photographs of children treated in the Intensive Care Unit to their parents in a situation where the possibility of contact with the child is limited by the epidemiological situation?
2. How did you feel when you were offered this opportunity? What did you think then?
3. How did you feel when you received the first photographs of your child? What did you think subsequently?
4. How do you feel now that you receive regular photographs of your baby? What do you think now? OR How did you feel later when you received regular photographs of your baby? What did you think subsequently?
5. What can be done differently to better respond to the needs of parents?

### 2.6. Inclusion and Exclusion Criteria from the Study

Inclusion criteria: hospitalization of a child in the Intensive Care Unit of the University Children’s Hospital in Lublin for at least seven days, consent to participate in the study.

Exclusion criteria: mental state that makes it impossible to conduct an interview, e.g., severe symptoms of depression or anxiety, derealization; high risk of causing increased mental suffering or its worsening.

### 2.7. Setting

The interviews were conducted by telephone (12 interviews) and in person (two interviews). In the case of face-to-face interviews, they were held in a doctor’s office ensuring a sense of security and intimacy, without the presence of third parties. Interviews were done by telephone held in a place chosen by the participants and at a time convenient for them. Parents of 13 children, 13 women and four men participated in the study.

### 2.8. Data Collection

The interviews were not repeated. The interviews were audio-recorded. The researcher noted his observations and relevant information after the interview, but not during the interview. The duration of the recordings ranged from eight to twenty-two min, average fifteen min. The interview ended when all questions were answered. In the case of all of the participants, there were no indications to end the interview early, such as, e.g., severe mental suffering. The recordings were then transcribed. The call records were not submitted to the participants for authorization.

### 2.9. Data Analysis

Inductive coding was used, which is more accurate than deductive coding and gives a more complete, unbiased view of the subject in the research material. To the qualitative data analysis (QDA) computer software package NVivo (QSR International, Burlington, VT, USA) was used.

Audio recordings of the interviews were transcribed verbatim. Interview transcripts were read and compared with audio recordings for discrepancies. According to the chronology of the dates, the respondent was then assigned a number. The assigned numbers were used in the qualitative analysis. The parents were given an abbreviation where “M” meant mother and “T” meant father. Verbatim transcripts of respondents were read several times. The next step was to categorize notes by assigning topics. Material from each interview was included in the procedure. Thematic analysis was used, which is a useful method of exploring the perspectives of various research participants [9]. The topics were identified based on an analysis of parents’ data on: the usefulness of the intervention, parents’ emotional reaction to the received photos and potential ways to improve interventions.

## 3. Results

Seventeen parents participated in the study, including four males—fathers and thirteen females—mothers (Table 1). The average age of the respondents was 33 years. The median hospital stay was 13 days.

### 3.1. The Usefulness of the Intervention

Mothers who were sent photographs and videos of their baby reported that it was a useful tool for communicating about their baby’s health and development.

“*It is something fantastic, needed above all … since you started sending photos and videos of our son, I started to gain faith for the future, it is very, very needed, this is support. I have watched the video you sent me today dozens of times and I feel as if I was with her at that time. After all, even though the child is far from us, from me, I will have a piece of her, albeit a virtual one, with me*”

(M3)

“*In this situation, when you can’t go in there at all and see these children, it’s very cool*”

(M4)

“*I was very happy because it is a very good idea, a great idea, because a child changes from day to day, you just have some contact*”

(M2)

Parents’ statements indicate that mothers and fathers of newborns carefully look at the photos and look for signs of stabilization in the child’s condition and its development—favorable changes in the newborn’s appearance, weight gain, activity, less medical equipment, etc.

“*However, my baby is changing, that he has, I don’t know, more hair, that he is more pink and not red, that his hand is fatter…. I’m comparing one photo to another, so… well, that’s a great idea*”

(M7)

“*We were waiting for a photo with a pacifier, because these are the elements that show development*”

(M10)

Some parents believed that sending photographs and videos was standard practice in intensive care, but not all parents believed that it could be replaced by personal contact.

“*I thought it was like that in all wards, that it was standard in the hospital*”

(T2)

Parents felt that, during the pandemic, sending photographs helped them through this difficult period when they could not personally be with their child:

“*My husband and I are very happy with this idea, so for us it is really helpful and for us it is a great idea. Due to the fact that we can’t see each other, my husband has never seen Iga live, so at least in the pictures*”

(M5)

The photographs sent were passed on by the parents to other family members:

“*When I couldn’t call the doctor, and later I got these photos, it was calm… sometimes the photo is a bit misleading. In one photo, I don’t remember what day it was, but his hair came out very pale even though he doesn’t have any. Later, I sent them to my mother, to my husband, to all my friends, to my family*”

(M8)

Parents’ emotional reaction to the received photographs.

The parents’ responses to the proposal to send photographs of the child and their emotions while viewing the first photographs were strong and marked by significant ambivalence. Especially in the first period, the parents were accompanied primarily by difficult emotions, such as fear and grief.

“*When I knew that I had already received the photos from you, after a few days, I was stressed, I was simply afraid to see them…When I get such a photo or video, I just calm down in myself, because I know that everything is fine with her*”

(M1)

“*There was compassion and concern, a little longing*”

(M4)

“*The situation was very stressful for us, we were immediately emotional and crying……we were calmer*”

(M6)

“*This is both sadness and joy*”

(M9)

“*I went through a whole range of emotions. From regret, blaming everyone around*…”

(M12?)

Later, these emotions were largely replaced by emotions such as peace, joy, and emotion.

“*Emotion, joy, yes… emotion, joy that I have a souvenir*”

(M2)

“*The photos you send me, or videos, are watched non-stop and a smile on your face … appears every time, to be honest*”

(M3)

“*Emotion, of course, great …… we are waiting very much, we are waiting for the photos, we look at the post office when they will appear, so the emotion is certainly great and the expectation*”

(M5)

### 3.2. Potential Ways to Improve Interventions

While treating a child in an intensive care unit, nothing can replace direct parent–child contact, which should be prioritized. In the case of the COVID-19 pandemic, when the state of the epidemic led to the introduction into Poland of numerous legal consequences and restrictions, parents indicated that the regular sending of photographs of their newborn children was a significant support for them:

*“A sensational idea, because it would be difficult for us without it, it will not replace the parent’s visit on site, but it is some form of compensation (…) in fact, nothing can replace contact with the child, and this contact (…) is there really was something positive’’*

(M6)

“*I must admit that it is different when there is a personal contact than a photo contact, but it is very helpful. We realize that direct contact with touching and stroking is impossible to imitate. We didn’t expect it and [the photos] it was a bit of a surprise. Videos are better but the photos are good too*”

(M10)

Parents indicated that they would like to receive photographs of their child more often.

“*As for the photos you send, that’s something really big for me, because even that is twice a week. Of course, I would like to every day…*”

(M1)

It seems that it may be even more beneficial to send videos showing the child’s behavior or the possibility of “spying” on the child using a webcam:

“*Videos are better than photos, because you can see how this child moves, what faces he makes*’’

(M4)

“*A very good idea in these times…, cameras as if they were next to incubators, but I know that it is impossible to achieve such a thing*”

(M2)

In addition, one of the mothers appreciated the laconic information accompanying the electronic form of sending photographs on the appearance and/or behavior of the child on the day of sending the photograph:

“*However, this e-mail, or something like that in writing, is easier than someone telling me over the phone*”

(M7)

## 4. Discussion

Family-centered care and, more recently, family integrated care models have been adopted by neonatal intensive care units (NICUs) to encourage and empower parents to engage with and actively participate in the care of their infants, while collaborating with healthcare providers [10]. The experience of hospitalizing an infant is a traumatic event for new parents; additionally, it can be a time full of unpredictability, causing fear, anxiety and uncertainty in some people [11].

The COVID-19 pandemic has forced healthcare systems around the world to put in place preventive measures to reduce the spread of the virus. One recommendation was that “visitor numbers and visiting periods should be very limited” [7,12,13]. Pandemic visiting restrictions, stay-at-home orders, and physical distancing limited the ability of family members to be in person with infants in the neonatal ICU, making it difficult for family-centered care and leaving parents emotionally isolated from their newborns [10]. A study by Zorro et al. in the U.K. and the U.S. on parental perceptions of visiting restrictions during a pandemic showed that parents reported less involvement and less of a bond with their child [7].

### 4.1. The Usefulness of the Intervention

Staff support, in particular the essence of communication and bonds in the relationship between staff and parents, strengthens their positions and increases their safety in their parental role [7]. Virtual visitation (VV) in the NICU provides parents with instant access to their hospitalized infant via a one-way live webcam. This method is used more and more often; however, it still remains a new concept [14].

In our study, photographs and videos were recognized by the surveyed parents as an important complementary tool used in conjunction with staff support. From the studies of Gibson et al. the Babble app was seen by users as an essential adjunct to personally delivered information in the context of supportive relationships, and as a way to enhance relationships with other family members and friends, through the sharing of information [15]. In a study by Patel et al. Remote infant viewing (RIV) was used, enabling families to observe the newborn when there are visitor restrictions or when personal visits may be less private or not physically possible [12].

### 4.2. Parents’ Emotional Reaction to the Received Photos

With regard to the parents who took part in the study, we noticed that they were characterized by an attitude with a simultaneous positive and negative attitude towards the photographs or videos sent to them. At first, they were accompanied by anxiety, fear and uncertainty, and over time, joy, patience and emotion. Studies that measured emotions associated with stress, anxiety and/or depression in parents of children in ICUs indicate that approximately 25% of parents experience moderate to severe anxiety, and up to 50% report symptoms of depression while taking [16]. Knowing that parents of both genders experience anger, fear, loneliness and symptoms of anxiety and depression is important as nurses work with these parents. This storm of emotions is linked to stress responses and is associated with altered memory, low attentiveness, and ineffective verbal fluency. Nurses can anticipate the need for frequent information, replication, and assessment of parents’ knowledge of a critical situation as it develops [17]. According to the study by Aftyka et al., the source of stress in mothers of infants treated in ICUs is the loss of the parental role, as well as the child’s behavior and appearance, while the perception of the environment in which the infant is located appears to affect the level of stress the least [18]. A study by Meestres et al. showed that parents felt a large amount of stress related to the inability to be with their child. Parental stress related to ICU admission may have negative long-term consequences for parent–child interaction and child development [13].

### 4.3. Potential Ways to Improve Interventions

In our study, we found that mothers were interested in visits and contacted medical staff more often to obtain information about their newborn’s health. Similar results have been shown in studies by Rhoads et al. [19]. Nursing care can facilitate mother–infant bonding by encouraging communication [20]. The medical staff at our facility tried to help the mother to bond with her baby by providing support and encouragement by sending photos and videos. The respondents believe that films are a better solution because you can see the newborn as he or she moves at a given moment. According to research by Rhoads et al. webcam technology could be a potential intervention to help parents visit their newborns [19]. In a study by Mahoney et al. the use of a single-family ICU room design has been shown to best protect 24/7 parental presence after the emergence of COVID-19 [21].

## 5. Conclusions

This study showed how important it is to ensure communication between the parent and the medical staff. Despite the positive reception, in the future we should be consider obtaining consent from the legal guardian for taking pictures, whether this form will be accepted, and to ensure the presence of medical staff while the parent is watching the photographs/videos, as this method of communication will not fully ensure direct skin-to-skin contact to build bonds between the parent and the infant. Neonatal intensive care units need to find strategies to mitigate the impact of separation on parental experiences and bonds should a similar situation arise in the future.

## Figures and Tables

**Table 1:** Characteristics of the study group.

Patient No.	Reason for Hospitalization	Time of Stay in ICU (Days)	Interviewee/Kinship
P1	Extreme prematurity GA 23, severe asphyxia, extremely low body weight, circulatory failure, respiratory distress syndrome, sepsis	40	mother (M1)
P2	Down syndrome, respiratory and circulatory failure, congenital heart defect	14	mother (M2), father (T2)
P3	Respiratory and circulatory failure, respiratory distress syndrome	11	mother (M3)
P4	Enterocolitis necroticans, sepsis, retinopathy	14	mother (M4)
P5	Esophageal atresia	19	mother (M5)
P6	Severe asphyxia, respiratory and circulatory failure, congenital heart defect	12	mother (M6)
P7	Extreme prematurity 27 GA, extreme low birth weight, respiratory distress syndrome bronchopulmonary dysplasia, patent ductus arterosus, congenital pneumonia	38	mother (M7)
P8	Respiratory and circulatory failure, congenital pneumonia	16	mother (M8)
P9	Severe asphyxia, respiratory and circulatory failure, intracranial hemorrhage II grade, neonatal bowel obstruction	27	mother (M9)
P10	Prematurity 34 GA, seizures, intracranial hemorrhage IV grade	24	mother (M10)
P11	Severe asphyxia	60	mother (M11), father (T11)
P12	Prematurity 34 GA, seizures, intracranial hemorrhage IV grade	12	mother (M12), father (T12)
P13	Severe asphyxia	12	mother (M13), father (T13)

GA—gestational age.

## Data Availability

The authors declare that the data from this study are available from the principal investigator, Anna Aftyka.
